# Diagnostic Challenge: An Extremely Rare Case of Intra-Articular Myopericytoma of the Knee

**DOI:** 10.3390/diagnostics16101549

**Published:** 2026-05-20

**Authors:** Yoji Shido, Jiro Ichikawa, Mayu Fujihiro, Masanori Wako, Tetsuhiro Hagino, Kojiro Onohara

**Affiliations:** 1 Department of Orthopaedic Surgery, Hamamatsu University School of Medicine, Shizuoka 431-3192, Japan; shido@hama-med.ac.jp; 2 Department of Orthopaedic Surgery, Interdisciplinary Graduate School of Medicine, University of Yamanashi, Chuo 409-3898, Japan; wako@yamanashi.ac.jp (M.W.); t-hagino@yamanashi.ac.jp (T.H.); 3 Department of Pathology, Hamamatsu University School of Medicine, Shizuoka 431-3192, Japan; m.fuku@hama-med.ac.jp; 4 Department of Diagnostic Radiology, Interdisciplinary Graduate School of Medicine, University of Yamanashi, Chuo 409-3898, Japan; konohara@yamanashi.ac.jp

**Keywords:** myopericytoma, intra-articular, imaging, differential diagnosis, magnetic resonance imaging

## Abstract

We report an extremely rare case of intra-articular myopericytoma of the knee. A 66-year-old man presented with a 10-year history of knee pain and a slowly enlarging soft, tender, and elastic mass. Magnetic resonance imaging revealed a well-enhanced intra-articular lesion; however, the findings were nonspecific and raised a broad differential diagnosis of vascular, perivascular, and malignant soft-tissue tumors. Incisional biopsy followed by marginal excision confirmed the diagnosis of myopericytoma. Although typically benign and indolent, myopericytomas can mimic both benign and malignant lesions, necessitating histopathological evaluation. At 5 years after surgery, the patient remained recurrence-free.

**Figure 1:**
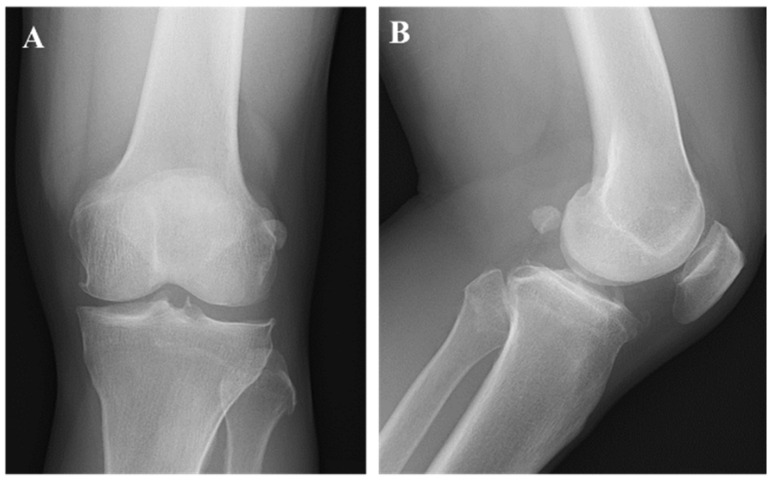
A 66-year-old man presented with a 10-year history of left knee pain. He had been diagnosed as having osteoarthritis of the knee at a local clinic and managed conservatively. However, over the past 5 years, the pain became more frequent, and he also noticed a palpable mass, prompting referral to our hospital for further evaluation. On physical examination, a soft, elastic mass was palpable around the left knee, with normal overlying skin color. Marked tenderness was present, but Tinel’s sign was absent. Joint effusion was noted, although there was no warmth. The range of motion of the knee was 0–120°, and despite pain, the patient was able to ambulate without assistance. His past medical history included surgeries for gastric and colorectal cancers, with no evidence of recurrence or metastasis at the time of our first evaluation. Laboratory tests indicated no elevation of inflammatory markers. Plain radiographs of the knee (**A**,**B**) demonstrated Kellgren–Lawrence grade 2 osteoarthritis, without soft tissue calcification or any obvious bony abnormalities.

**Figure 2:**
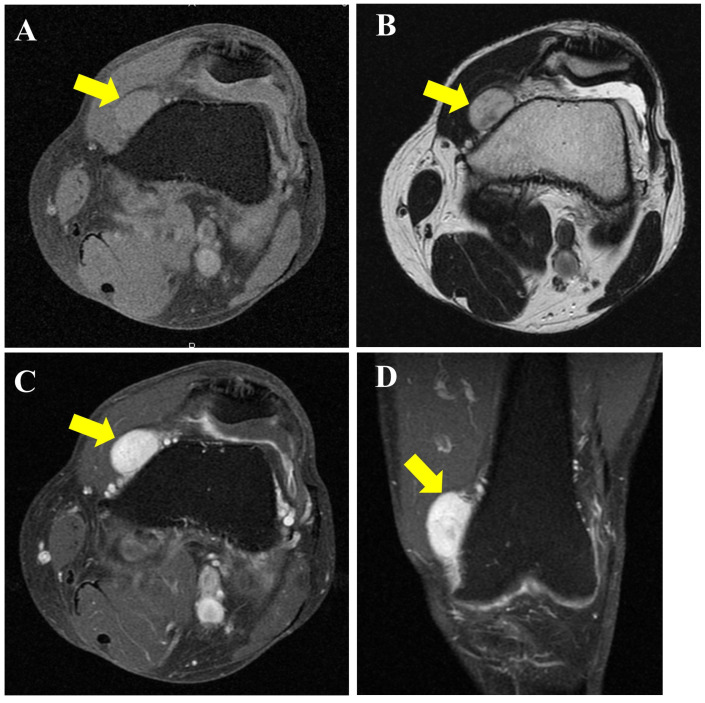
Magnetic resonance imaging (MRI) findings demonstrated an intra-articular lesion (yellow arrows). The tumor showed isointensity relative to muscle on T1-weighted fat-suppressed images (**A**), heterogeneous high signal intensity on T2-weighted images (**B**), and marked enhancement on contrast-enhanced T1-weighted images (**C**,**D**). Based on these MRI findings, the differential diagnosis included vascular and perivascular tumors (such as angioleiomyoma and myopericytoma), schwannoma, vascular malformation, and synovial sarcoma. To obtain a definitive diagnosis, an incisional biopsy was performed, which suggested a pericytic tumor, such as myopericytoma. The lesion was excised at the patient’s request to alleviate pain.

**Figure 3:**
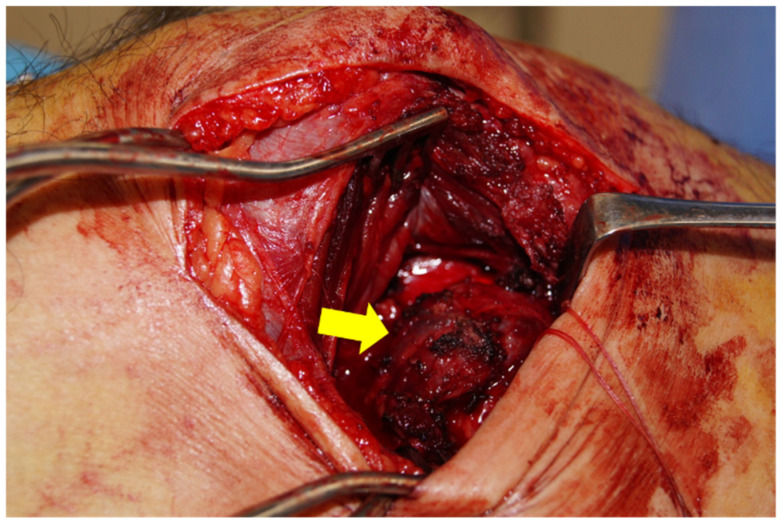
The previous skin incision was extended, and a portion of the vastus medialis was divided. Upon opening the joint capsule, the intra-articular tumor (yellow arrow) was visualized, and marginal excision was performed.

**Figure 4:**
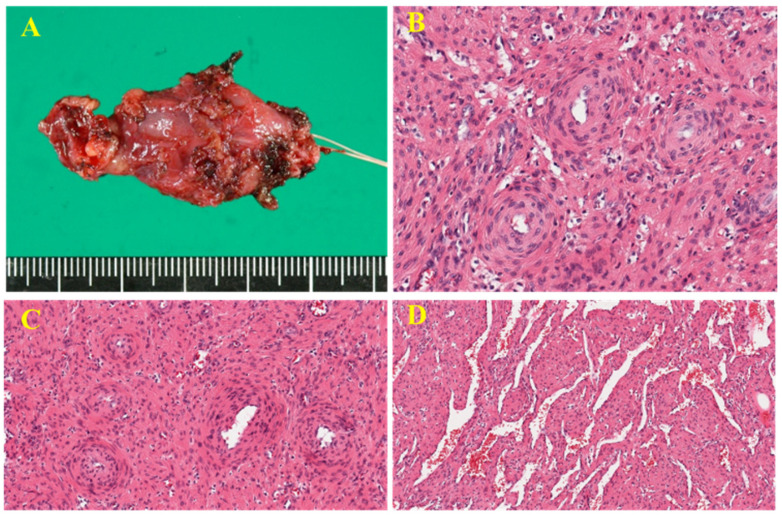
Macroscopically, the tumor was dark red and measured 30 × 20 × 10 mm (**A**). Histologically, oval-to-spindle-shaped tumor cells exhibited concentric perivascular proliferation, without mitotic figures or necrosis ((**B**) **×400**, (**C**) **×400**). A hemangiopericytoma-like pattern was also observed, characterized by numerous variably sized, branching, and dilated vessels ((**D**) **×200**). Based on these findings, a final diagnosis of myopericytoma was established. At 5 years after surgery, the patient remained recurrence-free. Myopericytoma is a perivascular myoid neoplasm and is considered a pericytic tumor, together with angioleiomyomas, glomus tumors, and myofibroma [1]. It most commonly occurs in the extremities, followed by the head and neck region and oral cavity; however, rare occurrences in visceral organs, intracranial locations, and bones have been reported [1,2]. Most tumors arise in the skin or subcutis, and deep-seated lesions are uncommon [3]. To the best of our knowledge, this is the first report of intra-articular myopericytoma of the knee. Clinically, myopericytoma is characterized by slight pain and slow growth, typically occurring in patients in their 40s and 50s, with a slight male predominance [1,3]. The average tumor size is approximately 2 cm, although lesions larger than 10 cm have been described [3]. In the present case, the tumor size, age and location were consistent with previous reports; however, the presence of marked tenderness was not typical. Myopericytomas often have a slow, indolent clinical course [1]. In the present case, nearly 5 years have passed since the mass was first identified. Although such an indolent course is generally suggestive of a benign tumor, it should be emphasized that certain sarcomas can also present with gradual growth and small-sized features that resemble those observed in this case [4]. Therefore, relying solely on the clinical course may lead to misdiagnosis, necessitating careful evaluation. Next, we discuss this case from the perspective of an intra-articular knee tumor. In general, most tumors arising in the knee joint are benign, and differential diagnoses commonly include tenosynovial giant cell tumors, synovial chondromatosis, and hemangioma. Tumor-like conditions, such as gout, amyloid deposition, rheumatoid arthritis, and ganglion cysts, should also be considered [5]. Although malignant soft tissue tumors are less common in this region, they must be considered in the differential diagnosis. In a series of 15 primary sarcomas of the knee joint, synovial sarcoma was the most frequently reported, followed by extraskeletal myxoid chondrosarcoma and myxofibrosarcoma [6]. Although rare, clear cell sarcoma [7] and myxoid liposarcoma [8] have also been reported. In our patient, hemangiomas and synovial sarcomas were initially considered differential diagnoses. Studies have indicated that myopericytomas often show low signal intensity on T1-weighted MRI images, high signal intensity on T2-weighted images, and strong contrast enhancement [2], similar to our findings. These features are also largely similar in angioleiomyomas and glomus tumors [9,10]. Additional MRI features that have been proposed include (1) the absence of peritumoral edema, (2) the presence of intratumoral vessels, (3) continuity or close proximity to adjacent vessels, and (4) internal nodules with low T2 signals. However, the sensitivity and specificity of these findings remain unclear [2]. In our patient, vessels surrounding the tumor were also observed. Nevertheless, because myopericytomas are often small, these characteristic MRI findings may not always be significant. Given these considerations, when MRI findings suggest multiple possible diagnoses, pathological confirmation through biopsy is appropriate. Histopathological evaluation is essential for diagnosing myopericytoma. Histologically, myopericytomas comprise oval-to-spindle-shaped myoid cells that proliferate in multilayered concentric arrangements around blood vessels [1]. However, the morphological spectrum is broad, and overlaps with those of other pericytic tumors, such as angioleiomyoma, myofibroma, and glomus tumors [3], thereby making differentiation challenging [11]. Mutations in PDGFRB and NOTCH3, once thought to be useful for diagnosing myopericytoma, are also relatively nonspecific, having been identified in angioleiomyoma and glomus tumors [11]. Although extremely rare, malignant myopericytomas have been reported; they are characterized by relatively rapid growth and high-grade histological features [12], including loss of circumscription and increased cellularity, pleomorphism, mitotic activity, and necrosis [12]. Because malignant myopericytoma may be associated with poor prognosis, caution should be exercised when making a diagnosis. The principle of treatment is surgical excision, and marginal resection is generally sufficient [2]. Although uncommon, recurrence has been reported, including one arising within a vessel [3], reflecting continued tumor growth rather than true recurrence. Radiotherapy has been used in cases of intraosseous myopericytoma with incomplete resection; however, its effectiveness remains unclear [13]. Because malignant transformation of a previously benign myopericytoma has not been reported, additional surgical excision is considered appropriate in cases of recurrence. This case highlights an extremely rare intra-articular presentation of myopericytoma of the knee. Given its nonspecific imaging findings and long indolent course, histopathological confirmation is essential for an accurate diagnosis.

## Data Availability

The raw data supporting the conclusions of this article will be made available by the authors on request.
